# Outer membrane protein A (OmpA) as a potential therapeutic target for Acinetobacter baumannii infection

**DOI:** 10.1186/s12929-020-0617-7

**Published:** 2020-01-18

**Authors:** Dan Nie, Yue Hu, Zhou Chen, Mingkai Li, Zheng Hou, Xiaoxing Luo, Xinggang Mao, Xiaoyan Xue

**Affiliations:** 1 Department of pharmacology, Fourth Military Medical University, Xi’an, China; 2 Department of Neurosurgery, Xijing Hospital, Fourth Military Medical University, Xi’an, China

**Keywords:** *A. baumannii*, AbOmpA, Infection, Therapeutic target, Multidrug-resistant

## Abstract

*Acinetobacter baumannii* (*A. baumannii*) is an important opportunistic pathogen causing serious nosocomial infections, which is considered as the most threatening Gram-negative bacteria (GNB). Outer membrane protein A (OmpA), a major component of outer membrane proteins (OMPs) in GNB, is a key virulence factor which mediates bacterial biofilm formation, eukaryotic cell infection, antibiotic resistance and immunomodulation. The characteristics of OmpA in *Escherichia coli* (*E. coli*) have been extensively studied since 1974, but only in recent years researchers started to clarify the functions of OmpA in *A. baumannii*. In this review, we summarized the structure and functions of OmpA in *A. baumannii* (AbOmpA), collected novel therapeutic strategies against it for treating *A. baumannii* infection, and emphasized the feasibility of using AbOmpA as a potential therapeutic target.

## Background

*Acinetobacter baumannii* (*A. baumannii*) causes hospital acquired infections (HAI), such as ventilator-associated pneumonia, bacteremia, urinary tract infections, meningitis, and surgical wound infections [1, 2], which leads to an increasing mortality in patients. Risk factors for these infections includes mechanical ventilation, usage of broad-spectrum antibiotics, ICU stay time and coma [3]. Statistically, about 1,000,000 people globally were infected with *A. baumannii* every year, and half of these infections were caused by multidrug-resistant (MDR) strains [4]. The mortality rate of *A. baumannii* infection in ICU was 45~60%, even reaching 84.3% when patients were infected with extensive drug resistance (XDR) *A. baumannii* [5, 6]. In 2017, Carbapenem-resistant *A. baumannii* (CRAB) ranked first in terms of threats to human health and urgency of developing relevant antibiotics, according to the drug-resistant bacteria list released by WHO [7].

However, colistin (CST) and tegacycline, the most commonly used antibiotics against carbapenemase-producing GNB, have become inefficient due to antibiotic resistance [8, 9]. In order to achieve clinical effectiveness, physicians had to increase the dose of CST and combine it with tegacycline [10]. Unfortunately, for bacteremia and serious respiratory tract infections caused by CRAB and XDR-*A. baumannii* respectively, combination therapy still did not work effectively [11, 12]. Facing the serious bacterial resistance, researchers have realized that it is urgent to develop new antibacterial strategies rather than rely on traditional antibiotics.

Generally, there are two ways to design novel antimicrobial agents. The one is to inhibit the production of essential substance for bacteria survival [13, 14]; the other is to inhibit virulence factors or antibiotic resistance genes of pathogenic bacteria in order to suppress pathogenicity or improve their sensitivity to antibiotics [15, 16]. However, inhibiting single essential component inevitably brings about great evolutionary pressure to bacteria and promotes the development of high-level drug-resistant strains [17]. Therefore, the novel intervention strategy targeting non-enssential processes is the key to overcome bacterial resistance [18]. For instance, inhibition of AbaI/AbaR quorum sensing systems with natural or synthetic inhibitor agent blocks the communication among bacteria and inhibits biofilms’ formation [19]. As a class of important virulence factors in bacteria, outer membrane proteins (OMPs) have attracted much more attention.

OMPs are a class of unique integral membrane proteins anchored in OM, whose β-barrel structures were formed by 8 to 26 strands. There are large, extend loops between the strands on the extracellular side and short loops on the periplasmic side. These characteristics give OMPs high stability in membrane and capability of fighting against extremely harsh environments [20]. Although different OMPs possess different sequences and functions, they share similar structure and biological properties [21]. OMPs of bacteria consist of even number strands, and importantly the function and stand shear number depend on their sequences. For example, as virulence related proteins, complement-binding protein OmpX in *E.coli* [22] and fibronectin- and heparin*-*binding protein Ail in *Yersinia pestis* [23] share comparable similar structure, but their sequence identity was lower than 45%. The diversity of OMPs sequence occurs at N terminal substantially more than C terminal, and the conserved β signal controls folding and correct assembly of OMPs [24].

However, up to now, the types of OMPs in *A. baumannii* have not been identified clearly, and only a few scattered reports were available, mainly including BamA, LptD, Omp33–36, OmpW. CarO and OprD. BamA itself as an OMP automatically can insert into OM and is responsible for the assemble of other OMPs [25]; LptD mediates the transportation of LPS to outer membrane (OM), loss of which can cause the accumulation of intermediates and fault location of LPS, and eventually lead to the disruption of bacterial membrane integrity [26]; Omp33–36 is a channel for the passage of water, which can induce apoptosis of host cells by activating caspase and regulate autophagy [27]; OmpW is a kind of hydrophobic porin existing in OM and cytoplasm, and plays an important role in modulating homeostasis of iron ion in bacteria [28]; CarO and OprD are related with resistance of carbapenem [29].

Among those OMPs of *A. baumannii*, OmpA is the most deeply studied virulence factor which plays key roles in regulating the adhesion, aggressiveness, and biofilm formation of *A. baumannii* and immune response of host. Overproduction of OmpA is an independent risk factor for the mortality rate of nosocomial pneumonia and bacteremia caused by *A. baumannii* [30]. Furthermore, the expression level of OmpA measured by qRT-PCR can be used as a rapid diagnostic index for antibiotic-resistant *A. baumannii*, by which the results are highly consistent with those through traditional MIC analysis [31].

This review overviewed the structure, function, and pathogenesis of AbOmpA, summarized therapeutic strategies targeting AbOmpA, and highlighted why AbOmpA is a potential target for the treatment of *A. baumannii*.

### OmpA structure and function

OmpA was first identified as a heat-modifiable protein in *Escherichia coli* (*E. coli*) in 1974 [32] and originally purified in 1977. Its molecular mass ranges from 28 kDa to 36 kDa [33]. OmpA family is a group of surface-exposed, porin proteins with high-copy number in Omps of GNB. N-terminal domain of OmpA is an antiparallel β-barrel structure consisting of eight transmembrane strands so as to be embedded in the outer membrane. The eight strands are connected by four long loops on the surface of outer membrane and three short turns in periplasmic domain forming globular C-terminal [34]. Even in a specific bacterial strain, the amino acid sequences of OmpA are various among multiple subclasses [35].

In recent years, with clarifying the native structure of AbOmpA, researchers have found that the amino acids of AbOmpA from a variety of clinical isolates are highly conserved (> 89%), while they are not homologous to human proteome [36]. By comparing various OmpA-like proteins, the two conservative amino acids, R286 and Asp271, which are located in C-terminal domain of OmpA, were identified. Both amino acids non-covalently bind to diaminopimelate amino acid (DAP), a component of peptidoglycan (PGN) [37, 38]. This interaction suggests that OmpA plays a key role in maintaining bacterial surface integrity. Generally, in GNB species, OmpA stabilize their structure by self-dimerization, which prevents the bending connections between β-barrel structure and periplasmic from being lysed [39].

### AbOmpA is essential for *A.baumannii* to adhere to and invade epithelial cells

**Fig. 1:**
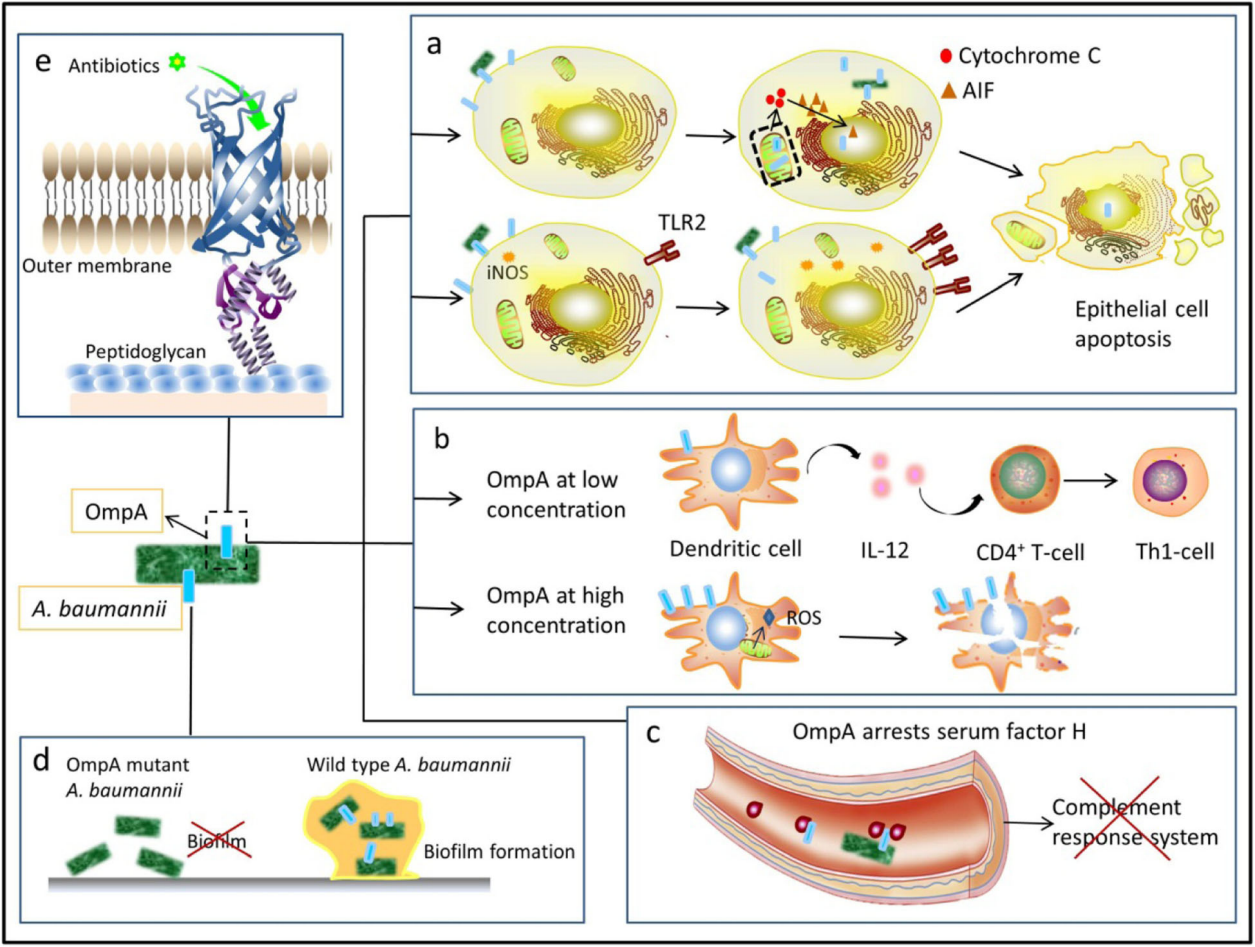
Functions of OmpA in *A. baumannii*. **a** upper panel. When contacting with epithelial cells, bacteria secrets OmpA into these cells. The OmpA are able to translocate in nucleus and mitochondria, and stimulate mitochondria to release cytochrome c. Then cytochrome c promotes apoptosis inducing factor (AIF) to translocate into nucleus and finally causes apoptosis of epithelial cells. Lower panel. OmpA increases the production of nitric oxide synthase (iNOS) and surface expression of Toll-like receptor 2 (TLR2) in epithelial cells, both of which trigger host cell death. **b** At low concentration, OmpA activates DCs which then stimulates CD4^+^T cells to exert Th1 response, while at high concentration; OmpA kills DCs by inducing mitochondria to release ROS. **c** AbOmpA can arrest serum factor H in serum, causing the paralysis of complement response. **d** AbOmpA play a dominant role in attaching abiotic surfaces and forming biofilm matrix. **e** AbOmpA is a porin protein which locates in outer membrane, selectively allowing the permeation of small molecular compounds

A. *baumannii* is capable of entering and persisting inside host cells. Firstly, it adheres to host cells, then invades and translocates into nucleus. After killing host cells, it disseminates in bloodstream and tissues [40]. AbOmpA mediates the adhesion and invasion of *A.baumannii* to epithelial cells [41](Fig. 1a, upper panel). Compared with wild-type bacteria, the isogenic AbOmpA-mutant strain is more difficult to invade host cells. Pre-incubation with recombinant AbOmpA (rAbOmpA) dramatically inhibits adhesion and invasion of highly invasive *A. baumannii* 05KA103 to epithelial cells. And in vivo, the pathogenesis is delayed by the mutation of AbOmpA, as evidence showed that less bacterial burden in blood of a murine pneumonia model [42]. For the adhesion mechanism of OmpA, Smani et al. [43] found that it was more easier for *A. baumannii* to attach the 96-well plates coated with fibronection than that coated with BSA, and they identified the protein binding with fibronection was OmpA, indicating that the binding of OmpA to firbronectin was the first step of the interaction between *A. baumannii* with host cells.

AbOmpA could directly cause host cells death if delivered to host cells via outer membrane vesicles (OMVs) [44]. As shown in Fig. 1a, after entering host cells, AbOmpA can localize to mitochondria, leading to the release of molecules (such as cytochrome C, apoptosis-inducing factor (AIF)) In early stage of *A.baumannii* infection, AIF degrades chromosome DNA and facilitates apoptosis of epithelial cells [45, 46]. In addition, OmpA can also translocate to nucleus of host cells depending on its monopartite nuclear localization signal (NLS)-“KTKEGRAMNRR”, which located between residues 320 and 330 of OmpA. This subcellular targeting of rAbOmpA can induce the apoptosis of host cells, but the specific underlying mechanism is not clear [46]. rAbOmpA purified from *A.baumannii* ATCC19606 at the concentration of 6 μg/mL is sufficient to exert cytotoxicity to human laryngeal epithelial HEp-2 cells [45]. AbOmpA promotes *A. baumannii* to adherent to and invade host cells, leading to their death by localizing in mitochondria and nucleus, but whether AbOmpA works in other organelles remains to be explored.

### AbOmpA stimulates innate immune response

AbOmpA also influences host immune system. Although AbOmpA treatment does not influence the expression level of pro-inflammatory cytokines or chemokine, it increases the production of nitric oxide synthase (iNOS) and surface expression of Toll-like receptor 2 (TLR2) in HEp-2 cells [47](Fig. 1a, lower panel). Both of them are important in host-defense mechanism. Nitric oxide (NO) exerts bacteriostatic and bactericidal functions in pulmonary infection [48], and this oxidative stress provides another causal factor to cell death. And TLRs recognizes pathogen-associated molecular patterns (PAMPs) and triggers immune response [49, 50]. The effects of AbOmpA on bone marrow-derived dendritic cells (DCs) are illustrated in Fig. 1b. AbOmpA at a concentration of 200 ng/mL activates DCs via TLR2, MAPK and NF-κB pathway, stimulating CD4^+^T cells towards a Th1 response [51, 52]. However, AbOmpA tends to kill DCs at high concentrations (≥3 μg/mL) by increasing the reactive oxygen species (ROS) from mitochondria [51, 53]. Inoculation with rAbOmpA could induce stype 2 immune response in mice, which damaged the balance between IL-4 and INF-c and led to the occurrence of infection [54]. In addition, AbOmpA paralyzed the complement response system by arresting serum factor H [55, 56] (Fig. 1c).

### AbOmpA induces biofilm formation

Biofilm enables *A.baumannii* to survive under hostile conditions [57], mainly consisting of protein, extracellular DNA and polysaccharides. According to the *statistics* of National Institutes of Health and the Center for Disease and Prevention, 65–80% human infections were caused by biofilm-forming bacteria [58]. Based on the multi-functional characteristics of biofilm formation, a series of genes associated with bacterial adhesion and biofilm formation were identified, such as *AbOmpA*, beta-lactamase PER-1 (*blaPER-1*) and biofilm-associated protein (*Bap*) [59]. Indeed, the OmpA of *A. baumannii* ATCC19606 plays a dominant role in forming stable biofilm on plastic surface (Fig. 1d). AbOmpA mutant strains fail to form biofilm, while replenishment of AbOmpA allele efficiently restores the ability [41].

### Permeation of small molecular antibiotics through AbOmpA

Porins are only β-barrel proteins that have been found in outer membrane of GNB. AbOmpA is the most abundant porin associated with drug resistance, epithelial cells attachment and biofilm formation [60, 61]. Smani et al. first demonstrated the effect of AbOmpA on the phenotype of multi-resistant *A. baumannii*, and found that the depletion of OmpA gene decreased the MICs of chloramphenicol, aztreonam, and nalidixic by 8, 8 and 2.67 fold, respectively. This data suggested that OmpA may participate in extrusion of antibacterial compound from periplasmic region and couple with efflux systems in inner membrane [62]. The transmembrane transport controlled by porins is an important way for delivering nutrients and small molecular hydrophilic antibacterial compound to bacteria [63]. Ramkumar et al. [64] found that AbOmpA selectively allowed the passage of small molecular antibiotics (Fig. 1e). For instance, ETX2514, the broad-spectrum β-lactamase inhibitor, penetrates through AbOmpA and enhances the antibacterial activity of sulbactam in an AbOmpA dependent way. In the future, clarifying AbOmpA crystal structure will reveal the relationship among preliminary structure of substrate, porin protein and permeation, providing more information for design of small molecular substrate [64].

### Regulation of AbOmpA expression

Among GNB, the factors influencing OmpA expression are mostly characterized in *Escherichia coli*, such as nutrients, culture conditions, bacteriophage infection and metabolic enzymes [65]. However, the OmpA-regulating mechanisms of *A. baumannii* are still being explored. Although OmpA in GNB possess a similar two-part structure, amino acid sequences located on the outer surface of bacteria are various among different genus [35]. Besides learning from the studies of *E. coli*, we are supposed to explore characteristics of OmpA in *A. baumannii.* In recent years, it has been shown that AbOmpA is a stress-related outer membrane protein, the expression of which is affected by internal and external environment of bacteria [66]. During studying the effects of temperature, dryness and nutrient deprivation on the long-term survival of sensitive strain ATCC19606 and clinical isolates, Bravo Z [67] et al. found that adhesion and biofilm formation related genes, *OmpA*, *bfmR* and *csuAB* were all down-regulated in starved cells, leading to difficulties in forming biofilms and spreading of *A. baumannii* in blood [67]. Hfq protein, a host bacterial factor first discovered in *E.coi,* is indispensable for RNA synthesis of bacteriophage Qb, [68], and now it is considered as a transcriptional regulator related to stress responses. Hfq deficiency retards cell growth and enhances cellular sensitivity to environment stress. The expression level of OmpA in Hfq mutant strain is dramatically lower than that in a wild type strain [69]. In addition, there is a causal correlation between biofilm formation and AbOmpA expression in *A.baumannii* under the treatment of antibiotics at sub-minimum inhibitory concentrations (MIC). Yoshinori et al. [70] incubated ATCC19606 and clinical isolates with 1/2 MIC of polymyxin B (PMB) and colistin (CST) for 24 h, respectively, and found that there was a positive correlation between the mRNA level of AbOmpA and the number of biofilm cells in CST treated ATCC19606 strain. The same results were observed in R3 clinical isolates in the presence of PMB [70]. Meropenem at the concentration of 64 μg/mL and 128 μg/mL increased AbOmpA expression by 1.81 and 1.63 folds, respectively [71].

Overall, there are three factors that mainly involved in the regulation of AbOmpA in recent studies. Firstly, disadvantaged environments such as starvation, decreases the production of AbOmpA, making it difficult for A. baumannii to form biofilm and adhere to host cells; Secondly, Hfq, a transcriptional regulator, whose expression level is closely related to that of AbOmpA; Finally, some antibiotics at sub-MIC promote AbOmpA expression and biofilm formation. Although several factors have been proved associated with OmpA exppresion in *A. baumannii,* the underlying mechanisms remain to be explored.

### Therapeutic strategies targeting AbOmpA

#### Polypeptide

Nowadays, the synthetic small polypeptide which specifically binds to OmpA has been designed to prevent *A. baumannii* from contacting with host cells. AOA-2, a cyclic hexapeptide as a blocking agent of AbOmpA without bactericidal activity decreases the adhesion of *A. baumannii*, *Pseudomonas aeruginosa* and *E. coli* to the surfaces of biotic and abiotic, and significantly enhances the sensitivity of *A. baumannii* to CST at the concentration of 125 μg/mL. In vivo, the intraperitoneal injection of AOA-2 (10 mg/kg) in combination with CST (10 mg/kg) improved the survival rate of mice with bacteraemia by 20% [72, 73]. In addition, some classic antimicrobial peptides (AMPs) interacting with AbOmpA have been gradually discovered, which are a series of endogenous defense peptides to kill bacteria and fungus [74–76]. For example, bovine myeloid antimicrobial peptide (BMAP-28) and its analog peptides killed MDR-*A. baumannii* by interacting with AbOmpA. These compounds started to destroy *A. baumannii* at the concentration of 40 μg/mL within only 15 min, and after 30 min, the bacterial cells were obviously damaged with leaking cytoplasm [77]. In addition, LL-37 interacted with the amino acid residues 74–84 of AbOmpA in a dose dependent way, decreasing the adhesion of *A. baumannii* to host cells. However, this inhibitory effect of LL-37 on adhesion was greatly reduced after AbOmpA deletion [78]. Human defensing-5 (HD5) is an endogenous peptide which kills MDR-*A. baumannii*. In order to enhance the antibacterial activity of HD5, non-cationic and non-hydrophobic residues were replaced by positively charged arginine to obtain a derivative named HD5d5, which could strongly bind to AbOmpA and then exerted its toxin-neutralizing function [79]. Although there is evidence that MDR*-Staphylococcus aureus* are resistant to LL-37 by producing catabolic enzymes [80], whether *A. baumannii* could develop resistance to natural AMPs has not been reported. The synthetic small peptide, specifically targeting AbOmpA without bactericidal activity, may avoid triggering bacterial evolution pressure and could be used alone or combined with other antibacterial compounds to exert synergistic effects.

#### Vaccine

As an ideal antigen of vaccine, AbOmpA is conserved among various clinical strains with the genome that is different from human genome [54, 81, 82]. Luo G et al. found that immunizing mice with 3 μg rOmpA in 0.1% aluminum hydroxide (Al (OH)_3_) significantly improved the survival rate of diabetic animals infected by *A. baumannii* HUMC1 by 40%, and decreased the counts of bacterial colonies of all the organs (except lung) by ten-fold, compared with control. In addition, the IgG antibody against rOmpA in serum was also dramatically enhanced [83]. Badmasti F et al. demonstrated that the survival rate of mice with disseminated sepsis induced by ATCC19606 was remarkably improved (70%) after treating with recombinant conserved immunodominant region of AbOmpA (8-346aa), and in addition, the combination with Bap(1-487aa) also increased the survival rate (> 80%) of mice infected by MDR AB-44 [84]. Furthermore, some derivatives of AbOmpA with higher antigenicity and lower toxicity were designed by bioinformatic and immunoinformatic tools. For example, a novel immunogenic model with 12 strands was obtained by modifying amino acid sequences of OmpA, in which K_320_ and K_322_ were substituted by Alanine, “NADEEFWN” was replaced by “YKYDFDGVNRGTRGTSEEGTL”, “VVQPGQEAAAPAAAQ” located at C-terminal and position 1–24 of N- terminal were removed. This AbOmpA-derived antigen was capable of triggering the production of antibodies which kills *Pseudomonas aeruginosa* and *A. baumannii* [36]. Another way is to develop DNA vaccine, which has attracted more attention owing to the effectiveness and durability. DNA vaccine is considerably safe and tolerable because it does not contain weakened or dead pathogenic during its production [85]. Hossein et al. cloned AbOmpA gene and inserted it into an eukaryotic expression vector pBudCE4.1 to obtain the recombinant pBudCE4.1–ompA. After transfected with this recombinant plasmid, human dermal fibroblast cells (HDF) effectively expressed AbOmpA [86]. Next, they evaluated the immunogenic potential of pBudCE4.1–ompA in mice model. After immunization with this vaccine, IL-2, IL-4, IL-12, IgM, IgG, and INF-γ all dramatically increased in serum and more animals survived compared to the control group [87]. However, we should not ignore the oncogenic potential of recombinant molecules due to its random integration into host genome.

#### Monoclonal antibodies (mAbs)

It is widely accepted that antibodies can be used to defense against microbial infections. Passive immunization induced by antibodies targeting AbOmpA used to be considered as a potential therapeutic method for MDR and XDR-*A. baumannii* infections [83]. However, treatment with polyclonal anti-OmpA sera has displayed many inevitable shortcomings, such as immune complex hypersensitivity, low content of specific antibodies and the potential danger of infectious diseases spread [88, 89]. Recently, monoclonal antibody (mAb) technology contributes to the development of antibacterial mAbs. Compared with polyclonal anti-OmpA sera, mAbs possess more advantages, such as higher safety, better homology and more specific targets [90]. The mAbs targeting OmpA promotes macrophages to kill *A. baumannii* 307.30 (AB307.30), except those covered with thick capsule, especially XDR-*A.baumannii.* Evidence showed that the binding of mAbs to clinical isolates was much weaker than that between mABs and ATCC19606. Whether the capsule over the cell wall prevents mAbs from binding to XDR-*A.baumannii*? The K1 capsule-negative mutant strain (AB307.30) strongly combined with Anti-OmpA mAbs, suggesting capsule polysaccharides may shield the binding sites of OmpA [91]. The poor combination between mAbs and XDR-*A. baumannii* needs to be solved in the further, and mAbs can also be used for other conserved epitopes of *A. baumannii*.

## Conclusions

With the emergence of MDR, XDR and pan-drug-resistant *A. baumannii* [92, 93], the traditional therapies have already failed to defeat complex infections caused by these pathogens. It is urgent to find new methods for treating drug-resistant *A. baumannii* infection. AbOmpA is becoming a potential therapeutic target for the following reasons. Firstly, AbOmpA is the most abundant and highly conserved porin in *A. baumannii*, which closely associated with virulence and bacteria survival under the hostile conditions. In addition, since it is not homologous to human genome, inhibition of it will not bring unnecessary damages to host cells. More importantly, AbOmpA is not essential for bacteria survival, suppression of which may not induce bacteria resistance. Although the studies about therapeutic strategies for *A.baumannii* infections are still limited and some even accompanied with certain risks, AbOmpA is a promising therapeutic target for the treatment of *A.baumanii* infections.

## Data Availability

Not applicable.

## Abbreviations

*A. baumannii*: *Acinetobacter baumannii*
AbOmpA: OmpA in *A. baumannii*
Bap: Biofilm-associated protein
blaPER-1: beta-lactamase PER-1
BMAP: Bovine myeloid antimicrobial peptide
CRAB: Carbapenem-resistant *A. baumannii*
CST: Colistin
DAP: Diaminopimelate amino acid
DCs: Dendritic cells
*E. coli*: *Escherichia coli*
GNB: Gram-negative bacteria
HAI: Hospital acquired infections
HD5: Human defensing-5
HDF: Dermal fibroblast cells
iNOS: Nitric oxide synthase
mAbs: monoclonal antibodies
MDR: Multidrug-resistant
MIC: Minimum inhibitory concentrations
NLS: Nuclear localization signal
NO: Nitric oxide
OM: Outer membrane
OmpA: Outer membrane protein A
OMPs: Outer membrane proteins
OMVs: Outer membrane vesicles
PAMPs: Pathogen-associated molecular patterns
PGN: Peptidoglycan
PMB: Polymyxin B
rAbOmpA: recombinant AbOmpA
TLR2: Toll-like receptor 2
XDR: Extensive drug resistance
